# Involvement of an Aberrant Vascular System in Neurodevelopmental, Neuropsychiatric, and Neuro-Degenerative Diseases

**DOI:** 10.3390/life13010221

**Published:** 2023-01-12

**Authors:** Keiichi Ishihara, Kazuyuki Takata, Ken-ichi Mizutani

**Affiliations:** 1 Department of Pathological Biochemistry, Division of Pathological Sciences, Kyoto Pharmaceutical University, Kyoto 607-8414, Japan; 2 Division of Integrated Pharmaceutical Sciences, Kyoto Pharmaceutical University, Kyoto 607-8414, Japan; 3 Laboratory of Stem Cell Biology, Graduate School of Pharmaceutical Sciences, Kobe Gakuin University, Kobe 650-8586, Japan

**Keywords:** neurodevelopmental disease, neurodegenerative disease, neuropsychiatric disease, vascular system

## Abstract

The vascular system of the prenatal brain is crucial for the development of the central nervous system. Communication between vessels and neural cells is bidirectional, and dysfunctional communication can lead to neurodevelopmental diseases. In the present review, we introduce neurodevelopmental and neuropsychiatric diseases potentially caused by disturbances in the neurovascular system and discuss candidate genes responsible for neurovascular system impairments. In contrast to diseases that can manifest during the developing stage, we have also summarized the disturbances of the neurovascular system in neurodegenerative diseases including Alzheimer’s disease and Parkinson’s disease. Furthermore, we discussed the role of abnormal vascularization and dysfunctional vessels in the development of neurovascular-related diseases.

## 1. Introduction

During the development of the murine central nervous system (CNS), the establishment of neural plates and the beginning of neural tube formation occur around embryonic day 7.5 (E7.5). Subsequently, dorsoventral patterning of neuroepithelial cells of the neural plate is established by E9.5 [1], with the proliferation of progenitors, differentiation into neural stem cells (NSCs), and migration of differentiated neurons and glia initiates to form the cerebral cortex in mice. CNS vascularization is initiated by the formation of the perineural vascular plexus (PNVP) at E8.5–10.5 in mice [2]. After the PNVP covers the CNS by E9, vessel ingression from the periventricular plexus into the cortex occurs at approximately E11.5 [3]. The association between embryonic NSCs and the vasculature thus appears necessary for CNS development [4,5]. According to this hypothesis, disturbances in brain vascularization during early life, including prenatal life, could impact brain formation through impaired neurogenesis. Conversely, impaired NSC characteristics reportedly influence brain vascularization, thereby suggesting that a close relationship between the nervous and vascular systems is essential for brain assembly [3,6]. In the present review, we introduce certain disorders that may be attributed to disturbances in brain vascularization.

In contrast to brain vascularization during early life, dysfunction of brain vessels may be involved in the development of cognitive decline. It has long been suggested that Alzheimer’s disease (AD), which accounts for a large proportion of dementia, is closely associated with cerebrovascular dysfunction [7,8]. In addition, cerebral amyloid angiopathy (CAA) has been detected in patients with AD [9]. Dementia with CAA alone is considered a form of vascular dementia, classified as AD when combined with significant AD pathology. Although there exists an overlap between AD and vascular dementia, and mixed dementia exists as well, this review focuses on the relationship between AD and cerebrovascular abnormalities. In addition to AD, vascular abnormalities in other neurodegenerative disorders, including Parkinson’s disease (PD), are discussed in our review.

The present review aims to shed light on the interplay between neurons and vascular system in neurodevelopmental, neuropsychiatric, and neuro-degenerative diseases.

## 2. Neural Stem Cells and Vascularization

During neural development, the vascular niche creates a specialized microenvironment via direct physical contact and secreted soluble factors.

In the embryonic neocortex, an avascular region without capillary vessel invasion is specifically constructed in the ventricular zone where mitotic NSCs are located, and NSCs transiently express HIF-1α, thereby attracting vascular endothelial tip cells. As a result, NSCs in contact with the pseudopodia of capillaries showed properties of undifferentiation, suggesting that direct contact with special ECs plays an important role in maintaining stemness [6]. Mitotic NSCs in the ventral telencephalon also induce vascular filopodia formation toward the ventricle in a cell cycle-dependent manner to regulate stem cell behavior [10]. In contrast, the adult subventricular zone (SVZ) is highly vascularized by a rich plexus of blood vessels [11]; however, the blood-brain barrier (BBB) in the SVZ has unique sites that have fewer glial endfeet and less pericyte (PE) coverage, allowing direct contact between NSCs and ECs [12]. In contrast, it is well established that soluble factors released from ECs regulate the behavior of NSCs, demonstrating the crucial role of the vascular niche in promoting the proliferation and differentiation of progenitors through soluble secreted cues [11,13,14,15]. Age-related changes in the vascular niche of the SVZ contribute to NSC depletion and dysfunction. For example, it has been suggested that the BBB in the SVZ is vulnerable and sensitive to age-dependent changes. Dividing NSCs are tightly juxtaposed with SVZ blood vessels during homeostasis and regeneration [12] as small circulating molecules in the blood enter the SVZ [12]. Recent evidence suggests reciprocal regulation between choroid plexuses and NSCs in a time- and region-dependent manner [16]. This is accomplished by region-specific secretion of molecules from each choroid plexus-cerebrospinal fluid (CSF) system as well as the competence of brain-region-specific NSCs to respond to the signaling molecules distributed in the CSF [16]. In addition, a recent study indicated that NSCs in the SVZ are particularly sensitive to age-related changes in the secretome of the lateral ventricle choroid plexus [17]. These findings suggest that the brain-specific capillary milieu influences NSCs expansion during development and deconstructs with age.

## 3. Neurodevelopmental and Neuropsychiatric Disorders Associated with Abnormal Vascularization

### 3.1. 22q11.2 Deletion Syndrome

The 22q11.2 deletion syndrome (22qDS; also known as DiGeorge’s syndrome) is caused by a 2.5-Mb hemizygous deletion of approximately 46 protein-coding genes on chromosome 22, including the Tbx1 gene [18]. Individuals with this syndrome are at high risk of neuropsychiatric disorders, including intellectual disability, schizophrenia, attention-deficit hyperactivity disorder, autism spectrum disorder, anxiety disorders, and seizures [19]. Based on neuroimaging studies, individuals with 22qDS exhibit reduced cortical thickness in specific brain regions [20,21], tortuous vessels, and subtle differences in cortical lamination [22], suggesting that neurogenesis and angiogenesis may be involved in the potential mechanisms underlying the neuropsychiatric phenotype of this syndrome.

**Figure 1:**
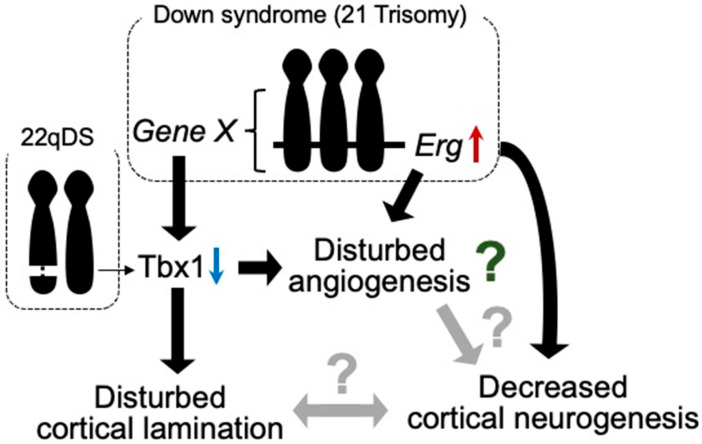
Tbx1 possibly relates to vascular abnormalities in 22qDS and DS. Triplication of Erg gene reduces prenatal cortical neurogenesis. ERG is dominantly expressed in endothelial cells and is suggested to play a role in angiogenesis. In DS, increased expression of certain gene(s) in Hsa21 decreases the expression of Tbx1. In 22qDS, a 3 Mb or a nested 1.5 Mb deletion of Hsa22q11.2 includes the TBX1 gene. TBX1 is suggested to play a role in cortical lamination and prenatal cortical neurogenesis. Thus, TBX1 may be involved in the molecular mechanism of common aspects in these syndromes, such as intellectual disability.

Among the hemizygously deleted genes in 22qDS, TBX1 has been implicated in neurogenesis and angiogenesis in animal models. Tbx1, a transcription factor participating in organ development during prenatal life, reportedly plays a role in brain angiogenesis [23] (Figure 1). Furthermore, Tbx1 heterozygous deficiency-mediated brain vascular anomalies such as brain vessel hyperplasia and increased filopodial density were recently demonstrated to be restored by Tbx1-Cre-induced activation of the vascular endothelial growth factor receptor 3 (*Vegfr3*) transgene, suggesting that the brain vascular phenotype caused by Tbx1 loss of function is associated with the dysregulated expression of Vegfr3 [24]. Tbx1-deficient mice were shown to exhibit abnormalities in brain ECs, along with enhanced angiogenic sprouting, resulting in an expanded vascular network [25]. However, the expanded vascular network is not functionally inactivated [26]. Tbx1^+/−^ mice showed the reduced proliferation of cortical progenitors and disturbed migration of glutamatergic cortical projection neurons and γ-aminobutyric acid (GABA)-mediated inhibitory neurons in the prenatal brain, with altered lamination documented in the adult cerebral cortex [25] (Figure 1).

Autism spectrum disorder (ASD) is a group of neurodevelopmental conditions characterized by early-onset dysfunctions in communication, impairments in social interaction, and repetitive and stereotyped behaviors and interests. Tbx1^+/−^ mice reportedly exhibit ADS-related behavioral phenotypes such as impaired social interaction, ultrasonic vocalization, memory-based behavioral alternation, working memory, and thigmotaxis [26,27], along with low fractional anisotropy signals, deficits in myelinated axons in the fimbria, and selectively delayed spatial memory acquisition [28], suggesting that Tbx1 is a gene responsible for the phenotypes of 22q11.2 hemizygosity-associated ASD. In addition, these mice demonstrated peripheral lymphatic vessel development via the Tbx1-mediated regulation of *Vegfr3* gene expression [29]. Based on these observations in Tbx1^+/−^ mice, disturbances in angiogenesis, lymphangiogenesis, and neurogenesis may be responsible for the psychiatric phenotypes in 22qDS, such as ASD (Figure 1).

In addition to Tbx1, claudin-5 (CLDN5) is also encoded in the region of hemizygous deletion in 22qDS. Interestingly, the frequency of schizophrenia is significantly elevated in 22qDS [30]. Furthermore, the CLDN5 variant rs10314, which is associated with a decreased claudin-5 expression, was detected in the remaining 22q11.2 region in 9 of 15 22qDS subjects with schizophrenia but in only 8 of 44 22qDS subjects without schizophrenia [31]. Cldn5-deficient mice exhibit size-selective leakage of the BBB to molecules with a weight up to 800 Da, despite displaying a normal development and morphology of cerebral vessels [32]. Therefore, CLDN5 is thought to play a role in the formation of the BBB. Taken together, these results suggest that BBB dysfunction caused by a deficiency of CLDN5 may lead to a high incidence of schizophrenia in 22qDS.

### 3.2. Down Syndrome (DS)

DS is a typical aneuploidy caused by the presence of an extra copy of human chromosome 21 (Hsa21). Individuals with DS exhibit numerous clinical features, including intellectual disability, developmental delay (growth retardation), characteristic facial features, and early onset AD-like dementia [33]. In autopsy studies, embryonic neurogenesis has been suggested to be decreased in human fetuses with DS compared with the non-DS population [34,35,36]. In addition, studies with mouse models of DS revealed the presence of decreased neurogenesis in the developing prenatal brain [37,38]. Several candidate genes that are associated with decreased prenatal cortical neurogenesis have been identified [39].

Although vascular malformations of the brain have not been reported in individuals with DS or mouse models of DS, several studies have demonstrated suppressed tumor angiogenesis in mouse models of DS [40,41]. These studies revealed that *Rcan1*, *Jam-b*, *Adamts1*, *Erg*, and *Pttg1lp* may be associated with the inhibition of tumor angiogenesis. In contrast to tumor angiogenesis, triplication of the *Erg* gene reduces cortical neurogenesis in the embryonic brains of DS model mice [42] (Figure 1).

Murine ERG is pre-dominantly expressed in mesodermal tissues, including the endothelial, precartilaginous, and urogenital areas, during embryogenesis [43]. ERG has also been suggested to play a role in the regulation of endothelial homeostasis, vascular development, and angiogenesis [44]. These findings indicate that *Erg* is one of the genes responsible for neurovascular abnormalities in developing brains with DS. In addition, we reported that Tbx1 expression is reduced in the brain of Ts1Cje mice, as well as in other mouse models of DS, during both prenatal and postnatal life [45] (Figure 1). Accordingly, Tbx1 may play a role in brain vascularization [23]. Using an inducible-X-inactive specific transcript method, silencing of Hsa21 in induced pluripotent stem cells (iPSCs) with DS showed that triplication of Hsa21 influenced the expression of genes related to neurogenesis and angiogenesis [46] (Figure 1). These reports suggest that anomalies in vascular development may be disturbed in prenatal brains with DS.

### 3.3. Schizophrenia

Schizophrenia is a complex neuropsychiatric disorder with an unknown etiology and poorly defined neuropathological and neurobiological features. Current genetic and neurobiological analyses have implicated neuronal developmental and synaptic plasticity abnormalities [47], neurotransmitters [48], microglia [49], and oligodendrocyte dysfunction [50] in schizophrenia. Alterations in prenatal brain development have been implicated as major risk factors for schizophrenia [51]. Imaging studies using postmortem brains of patients with schizophrenia have revealed abnormalities in cortical cell-type composition and macroscopic tissue organization, possibly stemming from aberrant brain development, such as a reduced density of parvalbumin (PV)-expressing GABAergic neurons in the prefrontal cortex [52], reduced cortical layer thickness [53,54], and enlarged lateral ventricles [55]. Human iPSCs with a mutation in the disrupted-in-schizophrenia 1 (DISC1) gene, which is a risk factor for a wide array of psychiatric illnesses, including schizophrenia, exhibit elevated WNT signaling activity with an altered expression of neuronal fate-related genes including an increased expression of dorsal progenitor markers and decreased expression of ventral progenitor markers [56,57]. Furthermore, in an organoid model of human iPSCs with a mutation in DISC1, a disorganized ventricular structure, decreased proliferation of neural progenitors, and disturbed formation of cortical layers 2/3 were observed [58]. Since these abnormalities are improved by a WNT antagonist, they seem to be caused by WNT signaling activation [59].

In addition, studies with iPSCs derived from patients with schizophrenia also suggest a disturbance in cortical neurogenesis with alterations in WNT signaling [60]. It has been shown that disturbed Wnt/β-catenin signaling affects cortical neurogenesis and ventricular morphogenesis in rodents, similar to the results obtained using iPSC models of DISC1 mutation [60,61]. In line with these observations, decreased WNT signaling activity has also been detected in iPSC-derived brain organoids from patients with schizophrenia [59]. Therefore, reduced WNT activity enhances differentiation into cortical GABAergic neurons with a reduced proliferation of NSCs [62].

Disruption of the BBB has been documented in schizophrenia, suggesting an association between BBB hyperpermeability and the pathogenesis of schizophrenia [63,64,65,66,67]. In an in vitro study using patient-derived ECs, human iPSCs consistently revealed an intrinsic failure in brain microvascular endothelial-like cells of patients with schizophrenia, thereby affecting proper angiogenesis and the BBB function, which may contribute to altered neurovascular crosstalk during schizophrenia [68]. Furthermore, epidemiological findings suggest that the risk of certain types of cancer, such as respiratory cancer, is significantly lower in patients with schizophrenia than in those without schizophrenia [69,70,71,72,73,74]. This reduced frequency of certain cancers may be attributed to the impairment of tumor angiogenesis. Accumulating evidence therefore suggests that abnormal vascularization in the brain with schizophrenia may impact brain development.

## 4. Neurodegenerative Disorders Associated with Abnormal Vascularization

### 4.1. AD and Cerebrovascular Abnormalities

In contrast to neurodevelopmental diseases, aging is a predominant risk factor for dementia. The incidence of dementia is estimated to double every 5.0–5.5 years in the over-65 population [75]. According to an estimation by AD International, approximately 130 million people worldwide will suffer from dementia by 2050. AD accounts for 60%–80% of all dementia cases. According to the amyloid cascade hypothesis, the accumulation of amyloid-β (Aβ) in the brain is the first trigger for the development of AD, followed by the formation of neurofibrillary tangles and synaptic and neuronal loss, which are pathological alterations directly related to cognitive decline [76]. Neurofibrillary tangles are formed by the intraneuronal accumulation of hyperphosphorylated tau protein. Aβ is generated from the Aβ precursor protein (APP) by sequential processing with β- and γ-secretases, followed by deposition into the brain parenchyma to form senile plaques in AD; this accumulation in the cerebrovascular space results in CAA formation.

**Figure 2:**
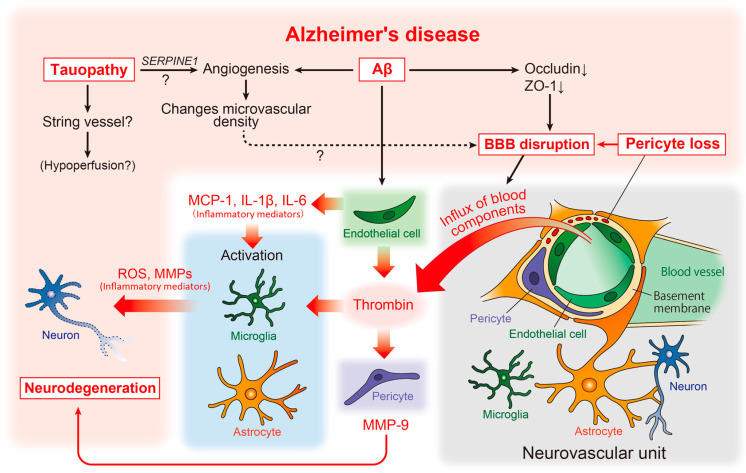
Neurovascular unit and cerebrovascular abnormality-related neurodegenerative pathways in AD pathophysiology. The relationship between vascular and brain component cells in the predicted AD pathology is depicted, including key neurodegenerative molecules. Aβ, amyloid-β; AD, Alzheimer’s disease; BBB, blood-brain barrier; IL, interleukin; MCP-1, monocyte chemoattractant protein-1; MMPs, matrix metalloproteinases; ROS, reactive oxygen species.

CAA has also been detected in the brains of patients with AD. As described above, a strong relationship between AD and cerebrovascular abnormalities has long been suggested. Although the pathogenesis of AD remains unclear, numerous accumulated reports suggest the involvement of the neurovascular unit [77], composed of cellular components such as neurons, astrocytes, microglia, pericytes, ECs, and smooth muscle cells, in the pathogenesis of AD (Figure 2).

At the macrostructural level, an altered microvascular density and numerous atrophic vessels are well established in the AD brain [78] (Figure 2). Enhanced microvascular density has been observed in the brains of an amyloid pathology mouse model (Tg2576 mice), as well as in human AD [79]. This enhancement was accompanied by increased angiogenesis and the disrupted expression of tight junction proteins such as occludin and zonula occludens-1 (ZO-1), suggesting that amyloidogenic events induce angiogenesis, which may affect the increased microvascular density and BBB disruption (Figure 2).

However, studies assessing the capillary density in human AD have shown conflicting findings, with some reporting an increase and others showing a decrease in capillary density [78]. This discrepancy may be explained by tauopathy. Recent studies in transgenic models of tauopathy (Tg4510 mice) have revealed that vascular density initially increases, followed by a decrease [80]. Moreover, the increased expression of angiogenesis-related genes such as *Vegfa*, *Serpine1*, and *Plau* was observed in ECs in the same mouse model, while the *SERPINE1* expression was elevated in human AD (Figure 2). Thus, Aβ and tau pathologies may independently affect vascular abnormalities, and the phenotype of vascular abnormality may vary according to the stage of AD. In the same study, aged mice with tauopathy showed an increased number of small-diameter blood vessels (<4 μm) lacking red blood cells, with adhered leukocytes often restricting downstream flow [80]. Small vessels may correspond to the “string vessels”, characterized by collapsed capillaries and dying ECs in the AD brain [81]. Further analysis of the effect of tauopathy on the formation of blood vessels may provide novel insights into the molecular mechanisms underlying blood hypoperfusion in the AD brain (Figure 2).

In contrast, VEGF binds to Aβ and is deposited in plaques in the brains of patients with AD, likely resulting in deficiency of available VEGF under hypoperfusion [82]. Moreover, the overexpression of VEGF in the CNS in a transgenic mouse model of AD significantly improved the integrity of the cerebrovasculature and functionally rescued mice from memory impairments [83], suggesting that VEGF can be effective in combating neurodegeneration and vascular dysfunction that occur during the progression of AD. In addition, lymphatic vessels are regulated by signaling between VEGF-C and its receptor VEGFR3, whereas impairments in this pathway lead to a deficiency in meningeal lymphatic vessels in the brain [84,85,86].

Reportedly, vascular abnormalities correspond to pericyte degeneration [87], and studies of postmortem brains have shown a correlation between pericyte loss and BBB disruption [88] (Figure 2). BBB disruption results in an influx of blood components such as thrombin into the brain parenchyma [89]. Thrombin activates microglia and astrocytes, increasing the release of neurotoxic reactive oxygen species and matrix metalloproteinases (MMPs), respectively [90,91] (Figure 2). Interestingly, the microvessels of patients with AD release significantly more inflammatory proteins, including thrombin and MMPs, than those of non-demented individuals [92]. Subsequent studies have revealed that thrombin synthesis is highly upregulated in ECs [93], whereas pericytes react most sensitively to thrombin and markedly increase MMP-9 production [94]. Furthermore, Aβ promotes the production of inflammatory mediators including monocyte chemoattractant protein (MCP)-1, interleukin (IL)-1β, and IL-6 [95] from endothelial cells. This suggests that, along with neurons and glial cells, vascular cells themselves may actively and directly contribute to the neurodegenerative process in AD [7] (Figure 2).

A significant decrease in acetylcholine synthase (ChAT) activity [96,97] and nicotinic acetylcholine receptors [98] has been observed in autopsied brains of patients with AD. Furthermore, cholinergic neurons in the nucleus basalis of Meynert in the basal forebrain have been shown to be impaired in the very early pathological stages of AD [99,100]. Thus, the profound involvement of cholinergic signaling disruption in the pathogenesis of Alzheimer’s has been recognized. These findings gave rise to the cholinergic hypothesis [101] and led to the development of acetylcholinesterase inhibitors (AChEIs) such as donepezil, galantamine, and rivastigmine [102]. However, these drugs are symptomatic treatments for AD with limited efficacy and duration. Consequently, there is a need to develop new disease-modifying therapies (DMTs) that can intervene in the pathogenesis of AD. However, the development of DMTs has been hindered by undetectable efficacy and the emergence of side effects in clinical trials. Therefore, at present, drug treatment for AD still relies on AChEIs [103].

The profound association between basal forebrain vasculopathy and cholinergic degeneration, which is detected early in AD pathology, and its involvement in AD pathogenesis have attracted much attention. Indeed, in humans, basal forebrain atrophy due to cholinergic neuron degeneration, altered cerebral blood flow, and exacerbation of Aβ lesions has been detected in parallel [104]. In a mouse model of AD, it was demonstrated that specific neurodegeneration of cholinergic neurons induced by murine p75NTR saporin (mu p75-SAP), a highly specific cholinergic immunotoxin, is associated with increased Aβ plaque deposition [105]. However, the association between the loss of cholinergic innervation, decreased vascular reactivity, and decreased clearance of brain Aβ has not yet been elucidated. Smooth muscle cells, which regulate arterial contraction and contribute to the regulation of cerebral blood flow in the brain, are innervated by cholinergic neurons that originate from the basal forebrain. ACh, released from cholinergic neurons, induces vasodilation by stimulating nitric oxide (NO) production, primarily through activation of endothelial nitric oxide synthase (eNOS). Thus, ACh contributes to regional arteriolar dilation and increased cerebral blood flow during neurovascular communication through eNOS activation. This suggests that eNOS is involved in AD pathology. In fact, decreased eNOS expression has been reported in the occipital cortex, which is hypoperfused in AD [106]. Furthermore, eNOS-deficient mice showed increased CAA levels without increased Aβ production [107]. However, one mechanism underlying ACh activation of eNOS is suggested to occur via the insulin-receptor substrate/PI3K/Akt pathway [108]. In line with this hypothesis, stimulation of the PI3K/Akt/eNOS pathway by fasudil hydrochloride, a selective ROCK inhibitor, increases cerebral blood flow [109]. Nizari et al. recently demonstrated the role of loss of cholinergic innervation in the onset and progression of CAA in a mouse model of brain Aβ pathology treated with mu p75-SAP. The results further indicated that the intramural periarterial drainage pathway via regulation of the vascular function by eNOS may be involved in the clearance mechanism in fasudil-treated mice [110]. These findings support the importance of the interrelationship between cholinergic innervation and the vascular function in Aβ accumulation in the brain. Furthermore, the data suggest that activation of the cholinergic neuron-eNOS axis may enhance the efficiency of Aβ removal from the brain.

Early-onset familial AD (FAD) represents less than 1% of all AD cases and is caused by a single genetic mutation of either *APP*, *PSEN1*, and *PSEN2* [111]. These mutations not only increase the production of Aβ but also vary the ratio of Aβ species to the aggregation-prone form (Aβ1-42) [76]. However, in the analysis of late-onset AD (sporadic AD), APOE ε4 was first identified as a risk gene. APOE ε4 is still considered the strongest genetic risk factor, including for early-onset AD. Furthermore, genome-wide association studies (GWAS) have identified more than 40 loci that are linked to AD risk and suggested that microglia are strongly implicated as the major cell type expressing GWAS genes [112]. Thus, there is a consensus that genetic factors have a strong influence on the development of AD, suggesting genetic overlap between AD and vascular pathology, primarily due to APOE [113]. However, little is known about the substantial association between genetic factors for AD and cerebrovascular abnormalities. One reason for this includes the difficulty of performing analyses due to the nature of blood vessels, which have abundant extracellular matrix around them and are rigid.

In this context, Yang et al. recently developed vessel isolation and nuclei extraction for sequencing (VINE-seq) to profile the major vascular and perivascular cell types of the human brain through 143,793 single-nucleus transcriptomes from nine individuals with AD and eight individuals with no cognitive impairment [114]. As a result, they demonstrated that at least 30 of the top 45 GWAS genes were enriched in cells of the human cerebrovascular system, again confirming the deep involvement of blood vessels and AD. They further found that GWAS genes predominantly expressed in microglia in mice, such as *APOE*, *CASS4*, *INPP5D*, and *HLA-DRB1*, were found to be strongly expressed in vascular cells in humans; this suggests the hypothesis that some AD risk genes and pathways may have been evolutionarily transferred from microglia to the vasculature from mice to humans. In addition, they also note that there is little overlap in the GWAS gene expression between mouse and human vascular cells, raising questions about the use of mice in studying cerebrovascular problems in human disease [114].

However, animal models, including mice, will be an essential tool for elucidating the mechanisms that substantially link genetic and pathological changes. Lee et al. identified *FMNL2* from a GWAS with 6568 AD cases and 8101 control subjects by interaction analysis with cardio and cerebrovascular risk factors [115]. Using the Aβ-injected zebrafish and transgenic mouse (APdE9 mice) models of AD, they further experimentally found that FMNL2 is upregulated and expressed in astrocytes with Aβ burden and loosens gliovascular interactions to promote Aβ clearance in brains. Thus, by identifying a stage- and cell-specific role of FMNL2, they suggest a compensatory function for this protein in AD pathophysiology and propose it as a target molecule for new drug development [115].

Appropriate interventions to address the risk of vascular dysfunction in daily life have been beneficial in reducing the prevalence of AD [116]. Understanding the molecular mechanisms underlying the pathophysiology of AD angiopathy is expected to lead to the development of DMTs and diagnostic strategies for AD.

### 4.2. Other Neurodegenerative Diseases and Cerebrovascular Abnormalities

Other neurodegenerative disorders, such as blood vessel alterations, BBB disruption, cerebral blood flow abnormalities, amyotrophic lateral sclerosis (ALS) [117,118], Huntington’s disease (HD) [119], and PD [120], are known. ALS is characterized by progressive dysfunction and degeneration of motor neurons. Perturbation of the BBB and the blood-spinal cord barrier (BSCB) has been observed in a mouse model of ALS [121]. The decreased expression of tight junction-related proteins, including ZO-1 and CLDN5, has been shown in the spinal cord microvessels of ALS model mice [121], indicating that brain/spinal cord vasculature dysmorphology and dysfunction may be involved in pathogenesis or disease progression.

HD, an inherited autosomal dominant neurodegenerative disease, is caused by the expansion of cytosine–adenine–guanine (CAG) repeats in the huntingtin gene. Accumulating evidence indicates that HD is associated with cerebrovascular changes, including increased microvascular density [122,123,124] and BBB dysfunction [122,125,126]. Cerebrovascular alterations may be involved in HD pathogenesis.

Vascular abnormalities in PD have been investigated and summarized previously. It has been suggested that increased angiogenic vessels in the brain regions are affected not only in patients with PD but also in rodent models of PD [127,128,129,130]. Newly generated vessels are immature and prone to BBB leakage, particularly when pericyte recruitment is impaired. Indeed, a dysfunctional BBB, resulting in high vascular permeability, has been demonstrated in a number of PD models [129,131,132,133] and individuals with PD [134,135,136]. A study using rubidium-82-PET consistently failed to detect BBB leakage in patients [136]. Thus, abnormalities in the vascular system may be a common pathogenic mechanism in a number of neurodegenerative disorders, such as AD, ALS, HD, and PD, and may represent a possible therapeutic target.

## 5. Conclusions

Anomalies in vascularization seem to be common in certain neurodevelopmental and neuropsychiatric diseases and neurodegenerative disorders. Interestingly, the expression of Tbx1, which is suggested to play a role in abnormal prenatal vascularization, is known to be disturbed in two developmental diseases, 22qDS and DS. Shared aspects of these syndromes, such as intellectual disability, may be associated with an abnormal Tbx1 function. An increased frequency of schizophrenia is observed in individuals with 22qDS [19]. There is a report examining whether or not the TBX1 gene is associated with schizophrenia, and results suggest that a couple of rare mutations in the TBX1 gene may contribute to the patho-genesis of schizophrenia in some patients [137]. In contrast to developmental diseases, BBB dysfunction and abnormal vascularization have also been implicated in AD. A high prevalence of early-onset AD has been detected in individuals with DS, with symptoms appearing before the age of 65 years old [138]. Furthermore, approximately 75% of individuals with DS over 60 years old exhibit clinical evidence of dementia [139]. The abnormal development of vessels in the brains with DS may contribute to the pathogenesis of early-onset AD. Thus, clarifying the role of abnormal brain vessels in neurodevelopmental disorders and neurodegenerative diseases may afford a new target for pharmacotherapy for these neurological diseases. Other factors, such as sex differences, should be considered in the vascular abnormality-based neurological diseases. Since the cerebral vasculature expressing several sex steroid receptors is a direct target of androgen, androgen therefore affects the cerebral vasculature, and it may be the foundation of sex differences in neurological diseases [140].

## Data Availability

Not applicable.
